# *Entropy* 2018 Best Paper Award

**DOI:** 10.3390/e21020130

**Published:** 2019-01-30

**Authors:** Entropy Editorial Office

**Affiliations:** MDPI, St. Alban-Anlage 66, 4052 Basel, Switzerland

On behalf of the Editor-in-Chief, Prof. Dr. Kevin H. Knuth, we are pleased to announce the Entropy Best Paper Award for 2018.

Papers published in 2017 were preselected by the Entropy Editorial Office based on the number of citations and downloads from the website. The winner nominations were made by a selection committee, which was chaired by the Editor-in-Chief and supported by twelve Editorial Board Members. The two top-voted papers have won the 2018 Entropy Best Paper Award and are shown below (in no particular order):

**Critical Behavior in Physics and Probabilistic Formal Languages**
Henry W. Lin and Max Tegmark
*Entropy* **2017**, *19*(7), 299; doi:10.3390/e19070299
Available online: https://www.mdpi.com/1099-4300/19/7/299

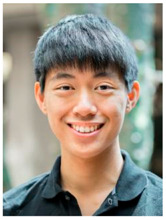

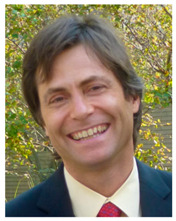
Henry W. LinMax Tegmark

Critical behavior, which describes the delay in long-range correlations as a power law with distance, is an interesting phenomenon that occurs in a wide variety of systems, physical or otherwise. We explore how natural language exhibits this phenomenon. Empirically, the mutual information decays in a similar way to a power law between symbols over a wide variety of texts. Theoretically, we analyze the decay of mutual information in various probabilistic models of natural language and find some broad criteria under which models may be expected to exhibit criticality. Our theorems are closely related to the results in statistical physics. Although the most naive models fail to reproduce power law correlations, more realistic models, such as context-free grammars, capture these correlations. Based on these ideas, we propose a simple quantitative test of any generative model of natural language. Interestingly, even modern recurrent neural networks with so-called long short-term memory (LSTM), which were designed to capture long-range correlations, struggle to ace our test.

**Multiscale Information Decomposition: Exact Computation for Multivariate Gaussian Processes**
Luca Faes, Daniele Marinazzo and Sebastiano Stramaglia
*Entropy* **2017**, *19*(8), 408; doi:10.3390/e19080408
Available online: https://www.mdpi.com/1099-4300/19/8/408

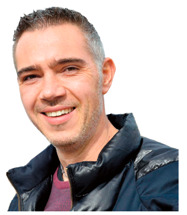

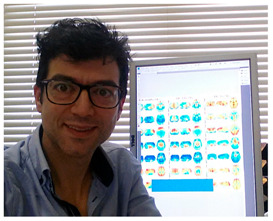

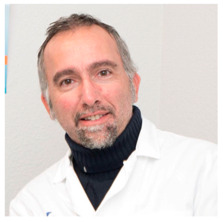
Luca FaesDaniele MarinazzoSebastiano Stramaglia

The dynamics of several real-world network systems in physics, neuroscience, climatology, biology, econometrics and other fields often arise from complex interactions among different subsystems. To study these interactions, information-theoretic approaches are often applied to the multivariate stochastic processes representing the system dynamics. In particular, information decomposition methods allow to quantify the entropy produced by each individual process or stored in it, the entropy transferred between processes and the synergetic or redundant amounts of entropy shared by two sources that transfer information towards a target process.

Moreover, a common trait of these network processes is that they typically exhibit dynamics spanning several temporal scales, which can range from milliseconds to thousands of years. In this context, our study addresses the fundamental problem of performing information decomposition across multiple time scales. This problem is currently unsolved because the change in the scale alters the causal relations between systems in a way that is still unknown. This is further complicated by the fact that the empirical approaches proposed so far to quantify causality for rescaled dynamics basically fail due to evident estimation issues. These issues actually constitute a bottleneck that needs to be settled in order to open the new research area of multiscale analysis of multivariate dynamic systems.

Our study serves exactly this purpose as it introduces the first theoretical framework that allows for an exact multiscale analysis of information transfer, redundancy and synergy in networks of multiple interacting stochastic processes. Under the assumption of linearity, we describe the process dynamics exploiting the state-space representation of multivariate autoregressive processes and derive exact expressions for the information that groups of source processes convey individually (unique information), redundantly (shared information) or only jointly (synergistic information) about an assigned output process.

These exact formulations result in a high computational reliability of the associated estimation framework, which is demonstrated in exemplary simulated systems. After this, the framework is applied to cortical neural signals measured in a patient with drug-resistant epilepsy. This illustrated that epilepsy is a network phenomenon associated with the emergence of information flowing from subcortical to cortical regions, wherein multiscale information decomposition helps to detect the localization of epileptogenic areas. Nevertheless, we emphasize the high generality of our approach, which is currently used to analyze climatological and physiological multivariate time series, and can be potentially applied to any context where multivariate time series can be measured from multiple interacting dynamic systems. Such a generality, together with the highly interdisciplinary nature of the topics treated in the work and with the availability of the algorithms implemented in a Matlab toolbox freely distributed with the article, will hopefully encourage the diffusion of multiscale information decomposition in a wide range of applicative fields.

 

**Prize Awarding Committee**

Entropy Editorial Board

